# The Analysis of NADPH Quinone Reductase 1 (NQO1) Polymorphism in Polish Patients with Colorectal Cancer

**DOI:** 10.3390/biom11071024

**Published:** 2021-07-14

**Authors:** Jarosław Gorący, Anna Bogacz, Izabela Uzar, Marlena Wolek, Małgorzata Łochyńska, Paweł Ziętek, Bogusław Czerny, Aneta Cymbaluk-Płoska, Piotr Modliborski, Adam Kamiński

**Affiliations:** 1 Independent Laboratory of Invasive Cardiology, Faculty of Medicine and Dentistry Pomeranian Medical University, 70-111 Szczecin, Poland; jargo@pum.edu.pl; 2 Department of Pharmacology and Phytochemistry, Institute of Natural Fibers and Medicinal Plants, 62-064 Plewiska, Poland; 3 Department of Pharmacology and Pharmacoeconomics, Faculty of Health Sciences, Pomeranian Medical University, 71-230 Szczecin, Poland; uzari@wp.pl (I.U.); boguslaw.czerny@iwnirz.pl (B.C.); 4 Department of Stem Cells and Regenerative Medicine, Institute of Natural Fibres and Medicinal Plants, 62-064 Plewiska, Poland; marlena.wolek@iwnirz.pl; 5 Institute of Natural Fibres and Medicinal Plants, 60-630 Poznań, Poland; malgorzata.lochynska@iwnirz.pl; 6 Department of Orthopedics, Traumatology and Orthopedic Oncology, Faculty of Medicine and Dentistry, Pomeranian Medical University, 71-252 Szczecin, Poland; pawelziet@gmail.com; 7 Department of Gynecological Surgery and Gynecological Oncology of Adults and Adolescents, Faculty of Medicine and Dentistry, Pomeranian Medical University, 70-111 Szczecin, Poland; aneta.cymbaluk@gmail.com; 8 Department of Orthopedics and Traumatology, Independent Public Clinical Hospital No. 1, Pomeranian Medical University, 71-252 Szczecin, Poland; modliborski.ortho@gmail.com (P.M.); emluc@wp.pl (A.K.)

**Keywords:** colorectal cancer, gene polymorphism, NQO1, polymerase chain reaction

## Abstract

Colorectal cancer (CRC) is one of the most common malignancies in Poland. Based on the findings of clinical trials, it is safe to conclude that genetic predisposition and environmental factors are the main factors responsible for the formation of colorectal cancer.The NQO1 gene plays an important role in reducing endogenous and exogenous quinones as well as quinone compounds to hydroquinones. It is an enzyme which is a part of the body’s antioxidant defense system. The aim of the study was to evaluate the correlation between the 609C > T polymorphism of the NQO1 gene and colorectal cancer risk in the Polish population. A total of 512 people were recruited for the study, including 279 patients with colorectal cancer, diagnosed at the University Hospital, Pomeranian Medical University in Szczecin. Genomic DNA was isolated from peripheral blood and the analyzed polymorphism was determined by PCR-RFLP. In the present study, we analyzed the clinical valuesand frequency of NQO1 609C > T polymorphism in patients diagnosed with colorectal cancer and controls. In case of the carriers of the TT genotype of the NQO1 polymorphism, an elevated risk for colorectal cancer was observed (OR = 2.96; 95% CI: 1.02–10.40). The analysis of the clinical parameters concerning the location and characteristics of the tumor stage revealed a statistically significant increase in the risk for colorectal cancer in the carriers of the TT genotype of the NQO1 polymorphism.

## 1. Introduction

Colorectal cancer is one of the most common malignant neoplasms in Poland, with a steadily increasing incidence amongst both men and women. Environmental factors and genetic predisposition have a large impact on the formation of colorectal cancer [1,2,3]. As far as the latter is concerned, mutations within protooncogenes, anti-oncogenes, and genes responsible for the process of apoptosis and DNA repair are notable. In addition, the activity of the enzymes involved in their biotransformation affects the carcinogenic effect of many exogenous factors. Changes in the genes encoding enzymes may predispose to cancer development or may have a protective effect [1,2,4].

Several studies have been carried out that utilize phase II detoxification enzyme genotypes (glutathione S-transferase (GST), UDP-glucuronosyltransferase (UGT), sulfotransferase (SULT) and N-acetyltransferase (NAT)) as molecular markers for cancer risk assessment. One of these enzymes, NADPH quinone 1 reductase (NQO1), which is involved in the detoxification of xenobiotics, including anti-cancer drugs, belongs to the DT-diaphorase family and demonstrates the highest activity in the liver, mainly in the cytoplasmic fraction, with much lower activity occurring in the kidneys, lungs, and brain. NQO1 plays an important role in reducing endogenous and exogenous quinones as well as quinone compounds to hydroquinones, and is a part of the body’s antioxidant defense system [4,5]. In addition, it is associated with protection against mutagenesis and carcinogenesis, because NQO1 plays a key role in the detoxification of various mutagenic compounds such as quinones derived from the diet or tobacco smoke [1]. NQO1 has a dimeric structure, consisting of 273 amino acids each, and is a flavoprotein containing the FAD prosthetic group. It is characterized by the presence of two protein isoforms which include two forms: hydrophobic and hydrophilic, with varying degrees of glycosylation. NQO1 catalyzes the two- or four-electron reduction of numerous endogenous and environmental quinone compounds to hydroquinones (e.g., ubiquinone, plastoquinone), which has a positive effect on the functioning of the body. The electron acceptor in reactions catalyzed by NADPH quinone 1 reductase may be NADH and NADPH [6]. The reductase gene or NQO1 has been shown to be polymorphic. In individuals with polymorphism in the NQO1 enzyme, it has been demonstrated that some allelic variants predispose to developing cancer. The 609C > T polymorphism of NQO1 results in non-synonymous mutation because is characterized by a change in the cytosine C gene to thymine T at position 609, which codes for proline and converts it to serine, consequently changing the amino structure of the enzyme. Patients may be homozygous for a particular allele, but they may also be heterozygous. This change in the spatial structure of the enzyme protein results in a decrease in FAD binding affinity and thus a loss of NQO1 activity [5,7]. Therefore, the aim of the study was to evaluate the correlation between the 609C > T polymorphism of the NQO1 gene and colorectal cancer risk.

## 2. Materials and Methods

A total of 512 subjects were recruited for the study, including 279 patients (105 women, 174 men; mean age: 62.49 ± 10.6 years) with colorectal cancer diagnosed and treated at the Clinical Hospital of the Pomeranian Medical University in Szczecin. Histological tests and endoscopic examinations were the basis for cancer diagnosis. The control group consisted of 233 patients (80 women, 153 men; mean age: 58.49 ± 10.6 years) included as healthy controls. The Local Ethics Committee approved of the study. All patients were informed about the purpose of the study and provided written informed consent.

Genetic analyses were conducted at the Department of Stem Cells and Regenerative Medicine, Institute of Natural Fibers in Poznan. Genomic DNA was isolated from peripheral blood leukocytes collected for EDTA. Before the DNA isolation, the material was stored at −20 °C. The commercially available “QIAamp DNA Blood Mini Kit” (Qiagen) was used for the DNA isolation. DNA isolation was carried out according to the manufacturer’s protocol (Qiagen, Valencia, Santa Clarita, CA, USA). DNA concentration was measured using a DeNovix DS-11 spectrophotometer (DeNovix Inc., Wilmington, NC, USA).

The analysis of the NQO1 gene polymorphism was performed using the PCR-RFLP (restriction fragment length polymorphism) technique, using specific digestion with a restriction enzyme DNA fragment. Restriction enzyme Hinf1 was used and the primers were used based on the results of Chen et al. [8]. The primer sequences used for the analysis of the 609C > T polymorphism of the NQO1 gene were as follows: F: TCC TCA GAG TGG CAT TCT GC, R: TCT CCT CAT CCT GTA CCT CT. The starters were synthesized at the Institute of Biochemistry and Biophysics, Polish Academy of Sciences, Warsaw, Poland.

**Figure 1:**
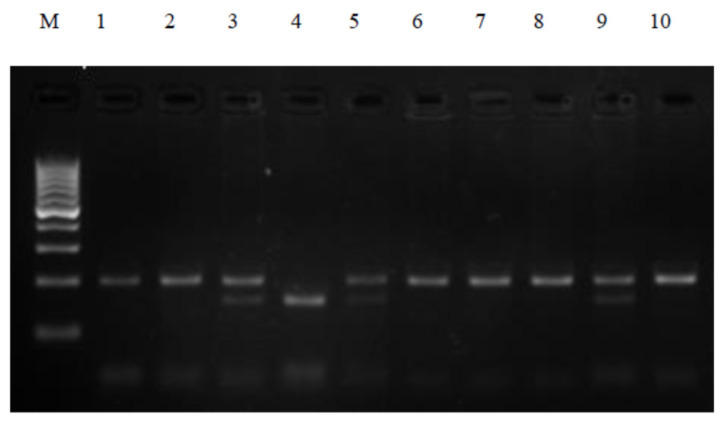
Restriction analysis of the 609C > T polymorphism in the NQO1 gene. Lane M—100 bp ladder marker; lane 1,2,6–8,10—CC genotype; lane 3,5,9—CT genotype; lane 4—TT genotype.

Polymerase chain reactions were performed in a thermocycler (PTC-200, MJ Research, Waltham, MA, USA) to ensure appropriate thermal conditions for the amplification of the selected sequences. The composition of the reaction mixture was determined experimentally. The PCR reaction was carried out in a 20 μL mixture containing 100 ng genomic DNA, 2 pmol each primer, 0.25 mM deoxynucleotide (dNTP), 1× concentrated PCR buffer, 2 mM MgCl2, and 1U Taq polymerase (Novazym, Poznań, Poland). PCR reaction conditions were as follows: pre-denaturation at 94 °C for 4 min, then 35 cycles: denaturation at 94 °C for 30 s, binding of primers at 65 °C for 30 s, elongation for 1 min at 72 °C, and final stage synthesis 5 min at 72 °C. PCR products were visualized on a 3% agarose gel stained with ethidium bromide for 40 min at 100 V. The electrophoresis products were evaluated using a documentation and computer analysis system UVI image (KS 4000/Image PC; Syngen Biotech Molecular Biology Instruments, Syngene, Frederick USA). The 230 bp PCR products were subjected to restriction analysis with the HinfI enzyme, followed by subsequent electrophoretic separation to visualize the DNA fragment. The CC genotype and the mutant TT genotype were defined as the presence of the 195 bp or 151 bp PCR product, respectively. Complete digestion of the 230 bp PCR product for the CT genotype gave fragments of 195 bp and 151 bp (Figure 1).

A statistical analysis of the results obtained was performed using the SPSS 17.0 PL program. The expected frequency of the genotypes was calculated using the Hardy–Weinberg equation, which was compared with the values observed using the chi-square test. The expected results were presented with a 95% confidence interval (P.U.). The correlations between the studied polymorphisms and the clinical parameters were also analyzed, and presented by providing the mean value, standard deviation, and standard error using one-way ANOVA analysis of variance.

## 3. Results

In the study group (*n* = 279), the most common histological types of colorectal cancer were as follows: adenocarcinoma with G2 differentiation (*n* = 205; 73.4%), adenocarcinoma G1 (*n* = 40; 14.3%), adenocarcinoma G3 (*n* = 16; 5.7%), carcinoma mucinosum (*n* = 12; 4.3%), and carcinoma gelatinosum(*n* = 6; 2.1%). The anatomical locations of the colorectal cancer in the study group were: sigmoid colon (*n* = 96; 34.4%), caecum and ascending colon (*n* = 78; 28%), rectum (*n* = 52; 18.6%), transverse colon (*n* = 39; 14%), and descending colon (*n* = 14; 5%). Postoperative histological examination, using the pTNM classification, revealed pre-invasive cancer (Tis) in 7 cases, in 40 patients the cancer did not cross the muscle membrane (T2), while in 153 cases the tumor crossed the muscle membrane and infiltrated the periocular tissues (T3). Tumor infiltration into the peritoneum (T4a) occurred in 68 patients, and in 11 cases it infiltrated neighboring organs (T4b). The absence of metastases to the regional lymph nodes was confirmed (N0) in 132 patients, whereas metastases in 1–3 lymph nodes (N1) and more than 4 lymph nodes (N2) were found in 89 and 58 patients, respectively. At diagnosis, metastases were found in 219 (M1), and not found (M0) in 61 patients. The tumor closed the intestinal lumen, causing gastrointestinal obstruction, in 44 cases, while in most cases (*n* = 235) there was no intestinal obstruction. Weight loss was a noticeable sign of the disease in 35 patients, but the vast majority of the subjects (*n* = 244) demonstrated no weight loss.

In addition, an analysis of the frequency distribution of the genotypes and the alleles of the C609T polymorphism of the NQO1 gene was performed in the study group and controls. There were no statistically significant differences in the distribution of the genotypes between both groups (*p* > 0.05). The dominant homozygotes (wild type, 609CC genotype) were most frequently observed in both groups. The expected and the observed values were comparable in both groups. The distribution was similar in the case of recessive homozygotes (609TT genotype) and heterozygotes (609CT genotype) (Table 1). An analysis of the frequency distribution of the C allele and the T allele of the NQO1609C > T polymorphism between the control group and the study group was performed. A similar relationship was observed in the frequency of the alleles in both groups (Table 1). An analysis of the impact of individual genotypes: CC, CT, and TT of the 609C > T polymorphism of the NQO1 gene on the risk for developing colorectal cancer was also performed. The risk factor (OR) was higher for the mutated TT genotype (OR = 2.96; 95% CI: 1.02–10.40) as compared to other CC genotypes (OR = 1.06; 95% CI: 0.72–1.56) and CT (OR = 0.77; 95% CI: 0.52–1.16) (Table 1). Hence, the TT genotype of the 609C > T polymorphism of the NQO1 gene may be associated with an increased risk of colorectal cancer and its metastasis. The effect of the C and T alleles and the 609C > T polymorphism of the NQO1 gene on the risk of colorectal cancer was analyzed. No statistically significant differences were observed (*p* = 0.33). Thus, no relationship between the C and the T alleles of the 609C > T polymorphism of the NQO1 gene and the risk of developing colorectal cancer was found.

In addition, the frequency of the CC, CT, and TT genotypes in the study group was also analyzed in relation to the obtained clinical data. The features of the clinical stage of the tumor were considered according to the pTNM classification and tumor location (Table 2).

## 4. Discussion

In Western Europe and the US, the incidence of colorectal cancer ranks third among all malignancies. This trend is associated with the impact of both environmental factors and genetic predisposition. The p-quinone reductase enzyme involved in the metabolism of carcinogenic compounds found in large numbers in the environment plays a special role in the development of colorectal cancer. NQO1 reduces the formation of free radicals which damage the cells. NQO1 reductase is also involved in the detoxification of xenobiotics and anti-cancer drugs with quinone structure (e.g., mitomycin, anthracycline). People who have a polymorphism in the NQO1 enzyme are presumed to have increased xenobiotic toxicity and, consequently, an increased risk for developing certain cancers.

The NQO1 gene, located on chromosome 16q22, is highly polymorphic [6]. The NQO1 609C > T polymorphism results from the substitution of thymine for cytosine at position 609 of the gene, which affects the enzymatic activity of NQO1 [9]. The 609C > T polymorphic variant may cause a decrease in the enzymatic activity of this gene. While analyzing the risk for developing various cancers, different polymorphic variants (homozygous TT variant, wild type CC, and heterozygous CT variant) were compared in individual ethnic groups.

Kesley et al. studied the incidence of homozygous NQOl polymorphism among various ethnic groups [9]. According to their reports, the frequency of the TT homozygotes of the NQO1 gene among African Americans is about 5%. Interestingly, 5% of Caucasians also had a homozygous polymorphism of this gene. The highest percentage (20.3%) of homozygous polymorphism of the NQO1 gene was observed in Asian populations, including 18.8% in Korean and 22.4% in Chinese populations. The incidence of heterozygous allelic variants in the abovementioned populations ranged from 34% to 52%. The results presented by Kesley et al, prove that the NQO1 gene polymorphism is a common phenomenon among the studied populations. Similar reports were published by Hu et al. [10]. Additionally, similar results were obtained by the Yu team, who investigated the incidence of the NQO1 gene polymorphism in different allele configurations between two ethnic groups. The incidence of TT, CT, and CC genotypes in the Caucasian population was 3.1%, 28.2%, and 68.7%, respectively, and 13.1%, 44.7%, and 42.2%, respectively, in the Asian population [11].

Endogenous and exogenous substances, accumulated in the body as a result of disorders of metabolic processes associated with polymorphism of genes responsible for detoxification, may have an impact on the formation of colorectal cancer. The literature offers reports on the relationship between the NQO1 609C > T polymorphism and colorectal cancer. The relationship in question is hypothetically associated with high levels of NQO1 expression in the small intestinal mucosa [7].

To date, various studies have been conducted to assess the relationship between the NQO1 609C > T polymorphism and the risk for colorectal cancer, but the results are inconsistent. A clinical study conducted among 685 patients (study group) and 778 healthy people (control group) confirmed that the NQO1 609C > T polymorphism is not associated with an increased risk of colorectal cancer in the Asian population [12]. However, another study by van der Logt et al., in 280 patients and 415 healthy people, suggested that the 609C > T NQO1 polymorphism was in fact associated with an increased risk of colorectal cancer in the Caucasian population [13]. Our results also confirm that the TT genotype of the 609C > T polymorphism of the NQO1 gene may be associated with an increased risk of colorectal cancer and its metastasis. As demonstrated in previous studies, the CC genotype has full enzymatic activity while the TT genotype shows null activity [14].

A recent study by Yu et al. also demonstrated a relationship between the 609C > T polymorphism of the NQO1 gene and the risk of colorectal cancer in the Caucasian population (CT + TT vs. CC, OR = 1.13, 95% CI = 1.00–1.28). However, this correlation was not observed in the Asian population [11]. 

Ding’s team obtained similar results in the Caucasian population [15]. The incidence of that polymorphism in that ethnic group was higher than in the Asian population (OR = 1.12, 95% CI = 1.04–1.21, *p* = 0.004 for TT + CT vs. CC). The abovementioned discrepancy suggests that the activity of the NQO1 enzyme depends on the characteristics of the study population, including age, sex, ethnicity, dietary factors, sample size, and exposure to carcinogenic environmental factors. These authors found that compared to the CC genotype, CT and TT genotypes were associated with an increased risk of CT colorectal cancer (OR = 2.02, 95% CI = 1.55–2.57); TT (OR = 2.51, 95% CI = 1.82–3.47), and that the risk for developing colorectal cancer doubles in patients with at least one allele variant. Their results clearly suggest that the T allele is associated with an increased risk for colorectal cancer in the Chinese population [15].

In another study by Wang et al. a relationship was observed between the 609C > T polymorphism of the NQO1 gene and the risk for developing colorectal cancer in different variants: CT (OR = 1.28, 95% CI = 1.08–1.51, *p* = 0.005); TT/CC: (OR = 1.60, 95% CI = 1.10–2.33, *p* = 0.015); TT/CT as compared to CC: (OR = 1.36, 95% CI = 1.09–1.69, *p* = 0.006); TT as compared to CT/CC (OR = 1.37, 95% CI = 1.05–1.80, *p* = 0.022) [16]. The subgroups were analyzed in both Caucasian and Asian populations. A similar distribution occurred in both groups, therefore a significant effect was found between the 609C > T polymorphism of the NQO1 gene and the risk for developing colorectal cancer [17].

Similar results were obtained by Zheng et al., who analyzed 14 clinical studies from various countries for the risk of developing colorectal cancer. The results of the analysis indicate that the NQO1 609C > T polymorphism was significantly associated with the risk of colorectal cancer (total OR = 1.30, 95% CI = 1.07–1.59) for CT vs. CC, 1.64 (1.15–2.33) for TT vs. CC, 1.34 (1.10–1.64) for TT/CT vs. CC, and 1.43 (1.10–1.87) for TT vs. CT/CC). Subgroup analysis showed that the T allele increased the risk for developing colorectal cancer in both Asian and Caucasian populations in case-control and hospital-based studies. In particular, the association of the 609C > T polymorphism of the NQO1 gene has been observed among smokers. The results of that meta-analysis suggest that the NQO1609C > T polymorphism significantly contributes to increased incidence of colorectal cancer in both Asian and Caucasian populations. 

In a study by Peng et al. [18], patients with histologically confirmed colorectal cancer were examined and the correlation between smoking and alcohol abuse and the 609C > T polymorphism of the NQO1 gene was investigated. It turned out that the occurrence of CT heterozygotes and TT homozygotes was associated with an increased risk for CT colorectal cancer (OR = 2.02, 95% CI = 1.55–2.57); TT (OR = 2.51, 95% CI = 1.82–3.47), as compared to wild-type CC. In addition, the 609C > T NQO1 polymorphism in chronic smokers and alcohol drinkers has been shown to have a greater impact on the risk for developing colorectal cancer than the polymorphism alone. These authors suggest that the 609C > T NQO1 polymorphism plays an important role in the development of colorectal cancer in the Chinese population. In addition, this effect is increased in case of smokers and alcohol users.

Leufkens et al. [19], Cleary et al. [20], and V. Harth [21] obtained similar results in their analyses and also reported a relationship between smoking and an increased risk for developing colorectal cancer in people with the NQO1 gene polymorphism.

NQO1 activity plays an important role in the metabolism of compounds contained in cigarette smoke such as nitrosamines, benzene, benzo (a) pyrene, and vinyl chloride [22]. Therefore, the 609C > T polymorphism of the NQO1 gene causes a decrease in the enzymatic activity of this enzyme, which may contribute to the intensification of the carcinogenic effect of the compounds contained in cigarette smoke on the risk for developing colorectal cancer.

In a study conducted by Hamajima et al. the frequency of the NQO1 gene polymorphism with variants (TT, CT, and CC) and the relationship with the incidence of different types of cancers was examined in a Japanese population [5]. Patients with lung, gastrointestinal tract (esophagus, stomach, colon), prostate, and breast cancers, as well as lymphoma (study group) and healthy controls, were included. The NQO1 gene polymorphism with the TT genotype was found in 12.7% of the patients with esophageal, 16.8% with stomach, 13.5% with colorectal, 9.7% with rectal, 17.7% with lung, 14.3% with breast, and 16.1% with prostate cancers and in 15.7% of patients with lymphoma. A comparison of the CC and TT genotypes revealed that patients with the CC genotype may more often develop lung cancer, and smokers with the TT genotype more often have esophageal and lung cancer. 

In another study of the Asian population, a relationship between the 609C > T oxidoreductase (NQO1) polymorphism and gastric cancer was found and a significantly increased risk (46%) of gastric cancer (95% CI = 1.20–1.79; *p* < 0.001) was demonstrated in people with the T allele [23]. Patients with the 609TT and the 609CC genotypes were compared and a strong correlation was observed (OR = 2.04, 95% CI = 1.37–3.05). These authors concluded that the 609C > T polymorphism of the NQO1 gene increases the risk for developing stomach cancer, especially in the Asian population [23]. Similar results were obtained by Liu et al. [24] and Zhu et al. [25]. Yang et al. also observed an increased risk of stomach cancer amongst Europeans [26].

## 5. Conclusions

Quinone oxidoreductase (NQO1) is an important enzyme involved in the detoxification of xenobiotics, and above all potentially carcinogenic compounds with the quinone structure. It is also credited with participation in the detoxification of anti-cancer drugs with a quinone structure (e.g., mitomycin, anthracycline). People who have a polymorphism in the NQO1 enzyme are presumed to have increased xenobiotics toxicity and, consequently, a higher risk of developing some cancers. In conclusion, our study demonstrated an increased risk for developing colorectal cancer in patients with the TT genotype of the NQO 609C > T polymorphism.Hence, it can be concluded that the NQO1 polymorphism is related to the risk of colorectal cancer and the process of metastasis in the Polish population.Future studies should be conducted in large-scale cohorts and should take into account the interactions of the genotype withotherfactorsconsidered(e.g., diet, tobacco smoke exposure).

## Figures and Tables

**Table 1:** Distribution of NQO1 609C > T polymorphism in patients with colorectal cancer and the controls.

Genotype	Patients		Controls				
	Observed Values *n* (%)	Expected Values %	Observed Values *n* (%)	Expected Values %	OR	95% CI	*p*
CC	187 (67.0)	64.8	153 (65.7)	66.9	1.06	0.72–1.56	0.41
CT	75 (26.9)	31.4	75 (32.2)	29.8	0.77	0.52–1.16	0.11
TT	17 (6.1)	3.8	5 (2.1)	3.3	2.96	1.02–10.40	0.02
Total	279 (100)	100	233 (100)	100	-	-	-
Alleles							
C	449 (80.5)	-	381 (81.8)	-	0.92	0.66–0.92	0.33
T	109 (19.5)	-	85 (18.2)	-	1.08	0.79–1.51	0.33
Total	558 (100)	-	466 (100)	-	-	-	-

**Table 2:** Selected clinical parameters of patients with colorectal cancer and the NQO1 609C > T genotype.

Clinical Parameters	Genotype *n* (%)			*p*
	CC	CT	TT	
pT				
Tis	10 (3.6%)	0 (0.0)	0 (0.0)	
T1	0 (0.0)	0 (0.0)	0 (0.0)	0.04
T2	12 (4.3%)	10 (3.6%)	0 (0.0%)	
T3	115 (41.2%)	50 (17.9%)	11 (3.9%)	
T4	50 (17.9%)	15 (5.4%)	6 (2.2%)	
pN				
N0	113 (40.5%)	28 (10.0%)	0 (0.0)	0.19
N1	40 (14.3%)	26 (9.3%)	10 (3.6%)	
N2	34 (12.2%)	21 (7.5%)	7 (2.5%)	
M				
M0	140 (50.1%)	60 (21.5%)	9 (3.2%)	0.03
M1	47 (16.8%)	15 (5.4%)	8 (2.9%)	
Tumor location				
•sigmoid colon	80 (29.7%)	16 (5.7%)	0 (0.0)	
•cecum and ascending colon	63 (22.6%)	15 (5.4%)	0 (0.0)	0.04
•rectum	28 (10.0%)	15 (5.4%)	9 (3.2%)	
•transverse colon	6 (2.2%)	25 (9.0%)	8 (2.9%)	
•descending colon	10 (3.6%)	4 (1.4%)	0 (0.0)	

Tis: carcinoma in situ, T1: tumor invades submucosa, T2: tumor invades muscularis propria, T3: tumor invades through the muscularis propria into pericolorectal tissues, T4: tumor penetrates to the surface of the visceral peritoneum, N0: no regional lymph node metastasis, N1: metastasis in 1–3 regional lymph nodes, N2: metastasis in 4 or more regional lymph nodes, M0: no distant metastasis, M1: metastasis to distant organs.

## Data Availability

Not applicable.
